# Lifetime-Dependent Effects of Bisphenol A on Asthma Development in an Experimental Mouse Model

**DOI:** 10.1371/journal.pone.0100468

**Published:** 2014-06-20

**Authors:** Susanne Petzold, Marco Averbeck, Jan C. Simon, Irina Lehmann, Tobias Polte

**Affiliations:** 1 UFZ – Helmholtz Centre for Environmental Research Leipzig-Halle, Department of Environmental Immunology, Leipzig, Germany; 2 Department of Dermatology, Venerology and Allergology, Leipzig University Medical Center, Leipzig, Germany; University of Southampton, United Kingdom

## Abstract

**Background:**

Environmental factors are thought to contribute significantly to the increase of asthma prevalence in the last two decades. Bisphenol A (BPA) is a xenoestrogen commonly used in consumer products and the plastic industry. There is evidence and an ongoing discussion that endocrine disruptors like BPA may affect human health and also exert alterations on in the immune system. The aim of this study was to investigate age-dependent effects of BPA on the asthma risk using a murine model to explain the controversial results reported till date.

**Methods:**

BALB/c mice were exposed to BPA via the drinking water for different time periods including pregnancy and breastfeeding. To induce an asthma phenotype, mice were sensitized to ovalbumin (OVA), followed by an intrapulmonary allergen challenge.

**Results:**

BPA exposure during pregnancy and breastfeeding had no significant effect on asthma development in the offspring. In contrast, lifelong exposure from birth until the last antigen challenge clearly increased eosinophilic inflammation in the lung, airway hyperreactivity and antigen-specific serum IgE levels in OVA-sensitized adult mice compared to mice without BPA exposure. Surprisingly, BPA intake during the sensitization period significantly reduced the development of allergic asthma. This effect was reversed in the presence of a glucocorticoid receptor antagonist.

**Conclusions:**

Our results demonstrate that the impact of BPA on asthma risk is strongly age-dependent and ranges from asthma-promoting to asthma-reducing effects. This could explain the diversity of results from previous studies regarding the observed health impact of BPA.

## Introduction

Asthma is a T helper 2 (Th2) cell–mediated immune response to common environmental allergens and is characterized by airway inflammation with pulmonary eosinophilia, airway hyperreactivity (AHR), and increased serum immunoglobulin E (IgE) levels [1]. Besides genetic predisposition, a strong contribution of environmental factors is reported to be responsible for the increase of allergic diseases in the last decades [2]. First hints from epidemiological studies suggest that amongst others the omnipresent endocrine disruptor bisphenol A (BPA) may be associated with the development of asthma and allergies [3]–[5]. BPA is commonly used in the plastic industry as well as consumer products like food containers, plastic bottles, thermal paper or dental fillings. Humans are exposed to this compound via ingestion, inhalation and dermal exposure throughout their entire life, including intrauterine life [6]–[8]. Therefore, the risk of BPA for human health has been discussed intensively in recent years. Previous data from epidemiological as well as animal studies suggest that BPA may affect the reproductive system, insulin production, mental and motor development, and the immune system [9], [10]. In particular, maternal exposure with its subsequent effects on disease risk in the offspring is of increasing interest since this period seems to be critical for the priming of the immune system [11]. However, the results from epidemiological studies and experimental animal models regarding the potential risk of BPA exposure on airway inflammation are highly diverse. Although some studies demonstrate asthma/wheezing-promoting effects of BPA [3], [4], [12], [13], inverse associations between urinary BPA levels and wheezing or even a Th1-increasing impact have also been reported [5], [14], [15]. Only some of the *in vitro* approaches provide data regarding the possible mechanisms for the immunomodulatory effects of BPA. The studies mainly describe an interference with hormone receptors like the estrogen receptor. However, these data are also inconsistent and show contrasting results [16], [17]. The inconsistency in the findings might arise due to different observation periods in human studies and the diverging design of the animal models, including the use of varying BPA doses and the route of exposure [18].

Since a direct causality between BPA exposure and an increased risk for asthma development cannot be studied in humans, we chose an experimental mouse model, which displays all hallmarks of the human disease, like eosinophilic inflammation of the lung, airway hyperreactivity and increased IgE levels [19]. Although differences in the metabolism of xenobiotics exist between humans and rodents, a similarity of BPA pharmacokinetics in women and mice was shown previously [20]. Therefore, in the present study we have investigated the effects of BPA exposure for different time periods during the whole life span on asthma development in an experimental mouse model. Our findings reported here, demonstrate that only a lifelong BPA exposure starting at birth exacerbated the allergic airway inflammation, whereas maternal exposure showed no effect on the disease outcome in the offspring. In fact, a BPA exposure of the adult mice even led to a reduced allergic immune response. These results imply that effects of BPA on allergic immune responses clearly depend on the time point of exposure during development and may range from asthma-suppressing to asthma-promoting impacts.

## Methods

### Mice

Female BALB/cByJ mice (6–8 weeks of age) were obtained from the Elevage Janvier Laboratory (Le Genest St Isle, France). Mice were bred and maintained in the animal facility at the University of Leipzig (Germany) under conventional conditions with 23°C room temperature, 60% humidity, and 12 h day/night rhythm. Control and BPA-exposed dams and pups were housed in polyphenylsulfone (PPS) cages (PPS can withstand very high temperatures without breaking down or releasing ingredient chemicals [21]) and bedded with LIGNOCEL bedding material. All mice received phytoestrogen-free diet (C1000 from Altromin, Lage, Germany) and water *ad libitum* from custom-built glass bottles to avoid contamination with BPA. Experiments involving an asthma induction included groups of 4–6 mice/cage and were performed at least 2 times according to institutional and state guidelines.

### Ethic statements

Animal protocols used in this study were approved by the Committee on Animal Welfare of Saxony/Leipzig (Permit Number: TVV10/10). Buffy Coats for isolation of peripheral blood mononuclear cells were obtained from healthy human donors (Institute of Transfusion Medicine, University of Leipzig, Germany) with fully informed written consent, conducted in accordance with the Declaration of Helsinki and approved by the Ethics Committee of the University of Leipzig (Permit Number: 135–2008).

### Exposure to BPA and OVA Immunization

BALB/c mice were exposed to BPA (5 µg/ml) exclusively via drinking water (custom-built glass bottles) for different time periods. Each exposure protocol was performed at least 2 times. To investigate the impact of an intrauterine exposure on the development of allergic airway inflammation in the offspring we exposed pregnant mice to BPA using our established transgenerational asthma model [22]–[24]. Briefly, female mice were exposed to BPA via drinking water one week before mating, though exposure was discontinued during the mating period of 1 week. Control dams received normal drinking water. Afterwards, BPA or normal water was given to pregnant mice either until delivery (prenatal exposure) or until weaning when pups were 3 weeks old (perinatal exposure). Just before delivery pregnant mice were separated into single cages. In a different approach mice were exposed to BPA via the same route starting after delivery during breast-feeding and offspring were further exposed during lifetime. Exposure of adult mice to BPA or normal water started one week before the first allergen injection and was continued until the end of the asthma induction protocol. To induce an allergic asthma the 6-week old offspring from at least 3 mothers (2–6 mice/group, at least 2x performed) were immunized intraperitoneally (i.p.) with the model allergen ovalbumin (OVA, 20 µg, Sigma-Aldrich, Steinheim, Germany) adsorbed to 2 mg of an aqueous solution of aluminum hydroxide and magnesium hydroxide (Alum, Perbio Science, Bonn, Germany) on days 1 and 14 followed by 20 µg OVA in 40 µl normal saline given intranasally (i.n.) on days 14–16 and 21–23 as described before [22], [24]–[28]. Control mice received Alum i.p. and normal saline i.n. To investigate a possible involvement of the glucocorticoid receptor (GR) 500 µg of the GR antagonist RU486 (Sigma-Aldrich) in 100 µl olive oil were injected i.p. 3 times per week during BPA exposure.

### Measurement of airway responsiveness

Lung resistance (R_L_) was measured by invasive plethysmography in response to inhaled methacholine (Sigma-Aldrich) as described previously [25], [28]. Therefore, mice were anesthetized (100 mg/kg ketamine and 10 mg/kg xylazine, Bayer, Leverkusen, Germany), intubated, and mechanically ventilated at a tidal volume of 0.2 ml and a frequency of 150 breath/min. Baseline R_L_ and responses to aerosolized saline (0.9% NaCl) were measured first, followed by responses to increasing doses (2.5 to 40 mg/ml) of aerosolized methacholine.

### Collection of bronchoalveolar lavage (BAL) fluid

All cells within the lavage fluid were counted using a hemocytometer. Diffquick (Medion Diagnostics AG, Düdingen, CH) stained cytospins were differentiated into eosinophils, macrophages, lymphocytes and neutrophils according to morphological criteria as described previously [22], [29].

### Lung histology and computer-based quantification of inflammation

Left lung was fixed in 10% formalin and stained with Haematoxylin & Eosin (H&E, MERCK, Darmstadt, Germany). For quantification and objective evaluation of the degree of histological inflammation, lung sections were scanned with a digital camera (Zeiss, 5 shots per lung) and analysed with HistoClick-Software based on morphometric image analysis [25], [30]. The degree of inflammation is expressed as the percentage of pixels which correlate to the stained cells of interest.

### OVA-specific IgE assay

OVA-specific IgE serum levels were measured by sandwich ELISA according to a standard protocol as described previously [25].

### Cytokine production

One day after airway function test splenocytes or mediastinal lymph node cells (5×10^6^ cells/ml per well) were isolated and re-stimulated *in vitro* with 200 µg/ml OVA in culture medium (RPMI medium supplemented with 10% FCS, 100 U/ml Penicillin, 100 µg/ml Streptomycin). After three days of culture cytokines were measured in supernatants using DuoSet ELISA kits (R&D Systems, Minneapolis, USA) according to the manufacturer’s instructions.

### BPA ELISA

BPA concentration in serum was detected with BPA Assay Kit (Immuno-Biological Laboratories, Hamburg, Germany). Serum samples, enzyme-labeled BPA and anti-BPA serum were added to a pre-coated microtiter plate with anti-rabbit IgG and incubated for 1 hour at room temperature. After washing, TMB was added as substrate and colour reaction was detected at 450 nm. BPA serum concentration was calculated from a standard curve with a detection range from 0.3–100 ng/ml.

### Culture and treatment of murine bone marrow-derived dendritic cells

Bone marrow cells were isolated from naïve mice and grown in RPMI medium containing 10% FCS and 20 ng/ml rmGM-CSF (Sigma) for 7 days. Some of the cells were cultured in the presence of BPA and maturation was induced by adding 1 µg/ml LPS (Sigma) for 24 hours at day 7 of culture. Cells were characterized by staining with an anti-CD11c–Phycoerythrin (PE) mAb. The supernatant was collected for IL-12 cytokine measurement according to manufacturer’s instructions (DuoSet ELISA kits, R&D Systems).

### Culture and treatment of human monocyte-derived dendritic cells

CD14^+^ monocytes were isolated with CD14 microbeads (Miltenyi Biotec GmbH, Bergisch Gladbach, Germany) from peripheral blood mononuclear cells of healthy donors and cultured in the presence of 100 U/ml IL-4 (PeproTech GmbH, Hamburg, Germany) and 1000 U/ml GM-CSF (Berlex, Richmond, VA, USA) for 5 days. Different BPA concentrations were added during the differentiation. To induce maturation 500 ng/ml LPS were added and IL-12 was measured in supernatants according to manufacturer’s instructions (IL-12 ELISA Ready-SET-Go!, eBioscience, Frankfurt, Germany).

### Statistical analysis

Mann-Whitney-U and Wilcoxon signed-rank test were used to determine statistical differences between groups. Data were expressed as mean ± SEM and *P* values of less than 0.05 were considered significant.

## Results

### Pre- and Perinatal Exposure to Low-dose BPA did not Affect the Asthma Outcome in the Offspring

**Figure 1:**
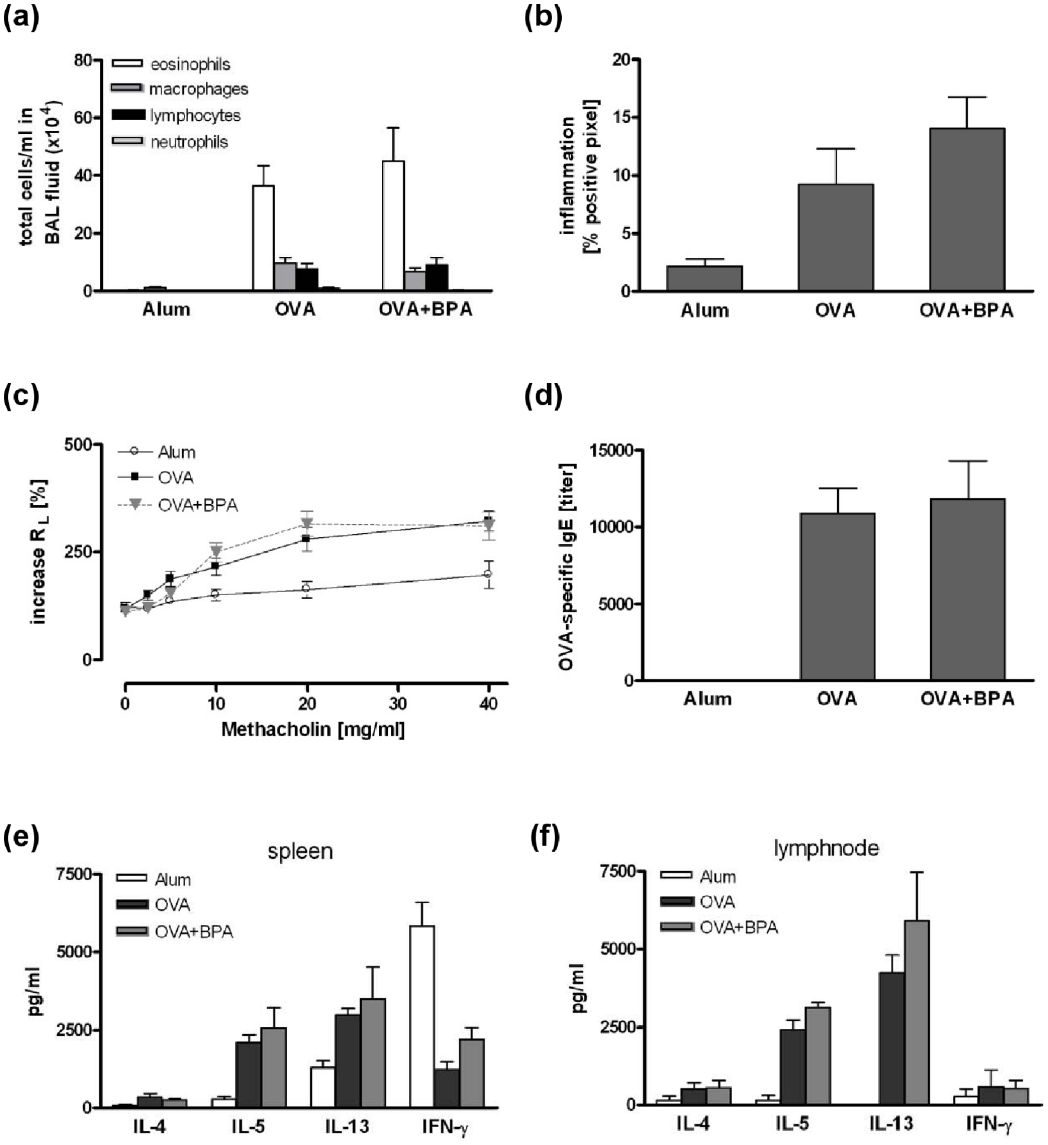
Prenatal BPA exposure did not affect the asthma phenotype in the offspring. Mice were exposed to 5 µg/ml BPA via drinking water during pregnancy. In the offspring an asthma phenotype was induced by sensitization to ovalbumin (OVA) followed by an intrapulmonary allergen challenge as described in Materials and Methods. BPA exposure did neither affect total cell number in BAL fluid (a), lung inflammation (b), lung resistance (c), OVA-specific IgE serum levels (d) nor cytokine production in splenocytes (e) or lymph node cells (f). Data are expressed as mean ± SEM, n≥11 animals per group.

**Figure 2:**
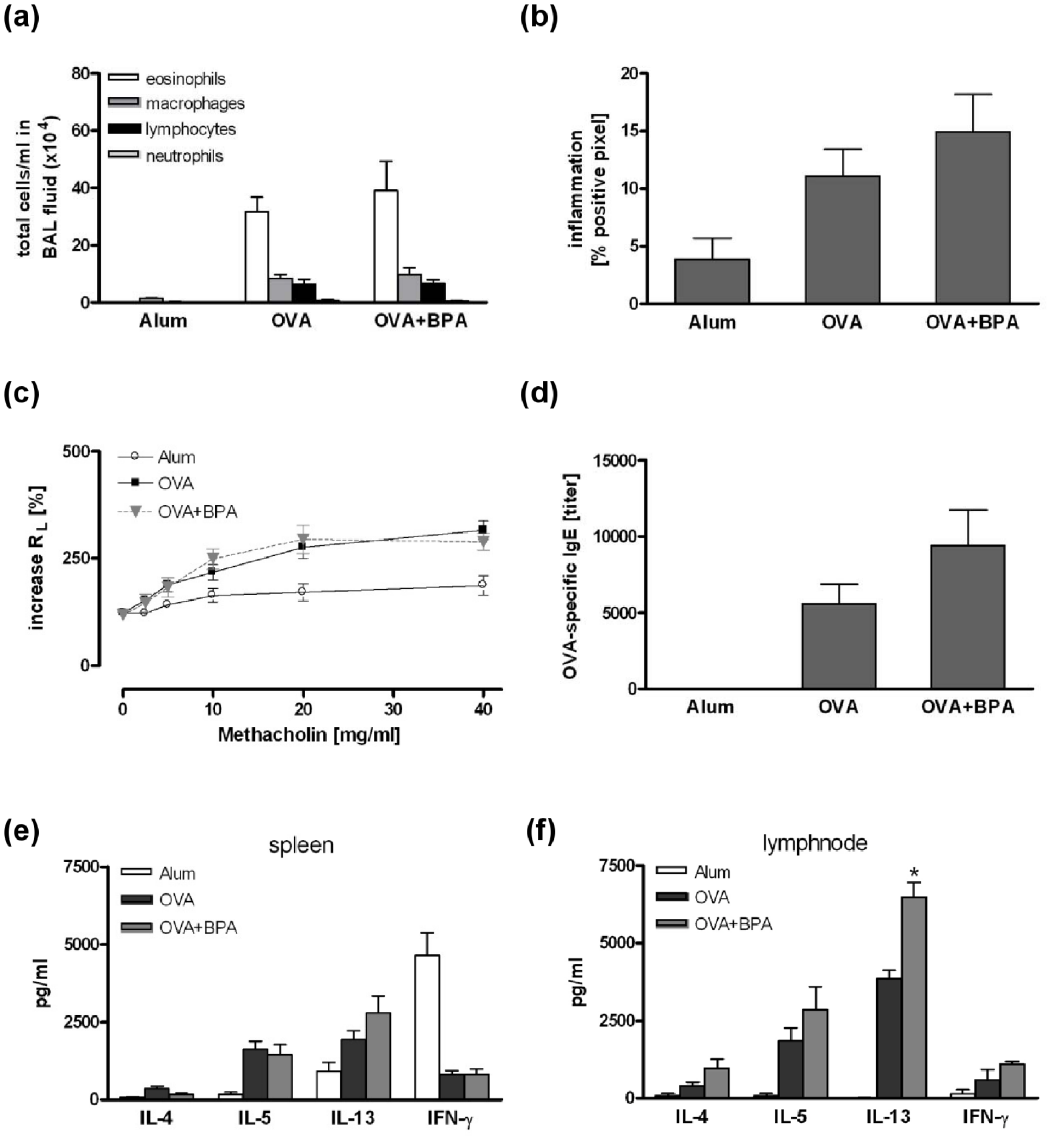
Perinatal BPA exposure showed no effect on the asthma phenotype in the offspring. Mice were exposed to 5 µg/ml BPA via drinking water during pregnancy and breastfeeding. In the offspring an asthma phenotype was induced by sensitization to OVA followed by an intrapulmonary allergen challenge as described in Materials and Methods. BPA exposure did neither affect total cell number in BAL fluid (a), lung inflammation (b), lung resistance (c), OVA-specific IgE serum levels (d) nor cytokine production in splenocytes (e) or lymph node cells (f). Data are expressed as mean ± SEM, n≥22 animals per group. *P<0.05.

OVA-sensitized offspring from three mothers exposed to BPA during gravidity did not develop an exacerbated allergic airway inflammation or an increased AHR compared to sensitized mice from three un-exposed mothers (Fig. 1A–F, offspring n≥11). Similar results were observed in offspring from dams exposed to BPA during both, gravidity and lactation (6 exposed or non-exposed mothers, offspring n≥22). A slight increase in lung inflammation, OVA-specific IgE levels and Th2 cytokine levels in the lymph nodes were observed, only IL-13 secretion was significantly increased (Fig. 2A–F).

### Lifelong exposure to BPA significantly increased the allergic airway inflammation

In the next experimental set-up three nursing mothers were exposed to BPA, whereas control mice (3) received only drinking water. After weaning the offspring (n≥6) were further exposed until the last allergen challenge. First, we measured BPA serum levels in adult mice as well as in their pups after an exposure for 21 days. BPA concentration reached 19 ng/ml in adult mice exposed to drinking water containing 5 µg/ml BPA. We could also detect BPA in the serum of the pups nursed by exposed mothers with a mean of 23 ng/ml (Table 1).

**Table 1:** BPA levels measured in serum.

BPA (µg/ml)^a^	Animals	*N*	Mean (ng/ml)	Range (ng/ml)
0	Adult	7	2.27±1.22	0.69–3.77
0.5	Adult	9	8.46±4.7**	3.21–15.83
5	Adult	13	19.17±9.12***	7.64–44.13
25	Adult	9	37.19±18.95***	19.02–79.51
0	Pup^b^	7	4.31±2.63	1.77–8.19
5	Pup^b^	7	23.99±7.26***	17.18–39.43

a BPA drinking water concentration.

b BPA levels were measured before weaning from pups of non-exposed and mother mice exposed during pregnancy and breastfeeding.

**P<0.01, ***P<0.001 compared to non-exposed control.

Mother mice were exposed during pregnancy and breastfeeding and adult mice for 5 weeks to BPA via drinking water. BPA serum levels of adult mice or pups were measured at the end of the exposure period as described in Methods.

**Figure 3:**
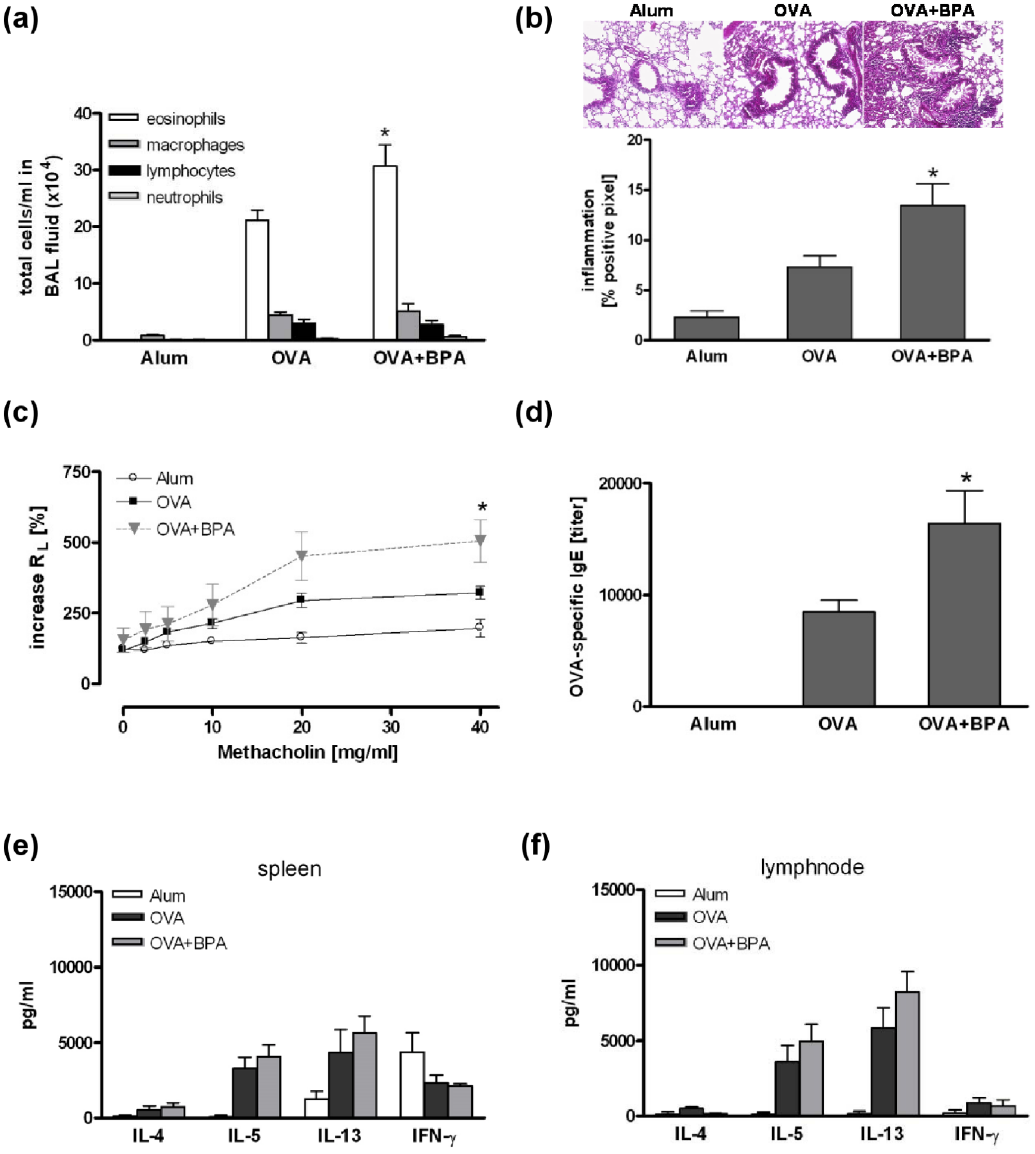
Lifelong exposure to BPA significantly increased the allergic airway inflammation. Nursing mice were exposed to 5 µg/ml BPA via drinking water and offspring during their lifetime. The asthma phenotype was induced by sensitization to OVA followed by an intrapulmonary allergen challenge as described in Materials and Methods. BPA exposure increased total cell number in BAL fluid (a), lung inflammation (b), lung resistance (c), and OVA-specific IgE serum levels (e). Cytokine production was not affected (e+f). Data are expressed as mean ± SEM, n≥6 animals per group. *P<0.05.

OVA-sensitized mice exposed to BPA as described above revealed an enhanced number of eosinophils in the BAL fluid compared to non-exposed control mice (Fig. 3A). Lifelong BPA intake also exacerbated airway inflammation in the lung (Fig. 3B) and AHR (Fig. 3C). Furthermore, serum levels of OVA-specific IgE were clearly enhanced (Fig. 3D), whereas we observed only a slight but not significant increase of Th2 cytokine levels in re-stimulated splenocytes and lymph node cells of BPA-exposed OVA-sensitized mice (Fig. 3E–F).

To examine whether BPA may affect the differentiation and function of dendritic cells (DC), which play a crucial role in processing environmental signals, we differentiated mouse and human DC from precursor cells *in vitro*. The presence of BPA during differentiation reduced the production of IL-12 after LPS-stimulation in both, murine and human DC (Fig. S1).

### Exposure of adult mice to BPA during sensitization reduced the allergic immune response

**Figure 4:**
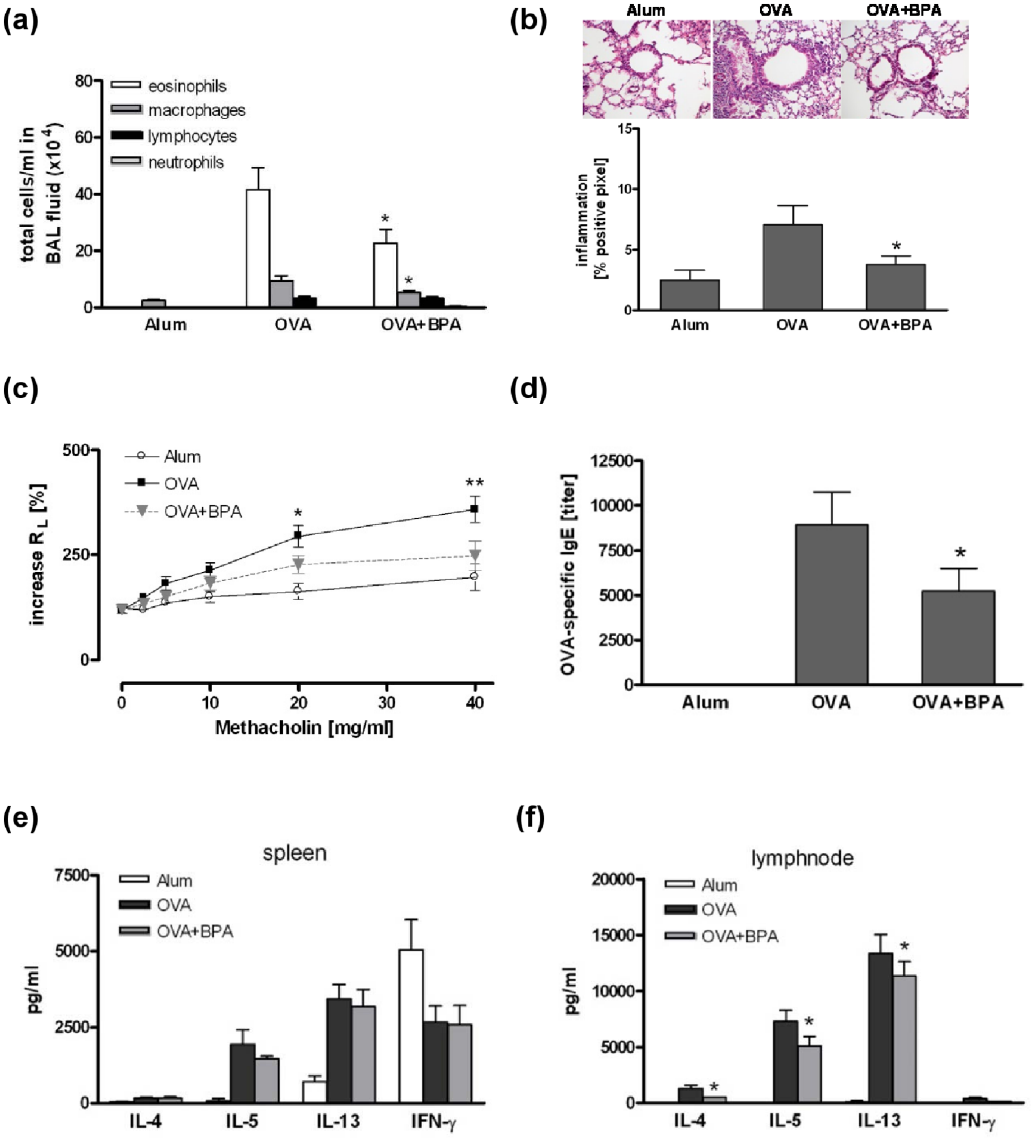
Exposure of adult mice to BPA during sensitization reduced the allergic immune response. Adult mice were exposed to 5 µg/ml BPA via drinking water during OVA-immunization. BPA exposure reduced total cell number in BAL fluid (a), lung inflammation (b), lung resistance (c), OVA-specific IgE serum levels (e) and Th2 cytokine production from lymph node cells (f), while cytokine production from spleen was not affected (e). Data are expressed as mean ± SEM, n≥18 animals per group. *P<0.05.

Surprisingly, BPA exposure of adult mice during OVA-sensitization diminished the number of eosinophilic granulocytes in the BAL fluid compared to non-exposed control animals (Fig. 4A). Accordingly, histological examination of H&E stained lung sections revealed a significantly decreased inflammation in the BPA-exposed group compared to control mice (Fig. 4B). BPA exposure also prevented the development of methacholine-induced AHR in BALB/c mice (Fig. 4C) and significantly reduced OVA-specific IgE levels compared to non-exposed OVA-sensitized control mice (Fig. 4D). In contrast, Th2 cytokine levels were only diminished in the lymph nodes but not in splenocytes (Fig. 4E,F).

To investigate whether different BPA exposure levels might induce alternate effects, we treated adult mice with two additional BPA concentrations (0.5 µg/ml or 25 µg/ml). BPA exposure to 25 µg/ml led to an even more reduced asthma phenotype in the OVA-sensitized adult BALB/c mice, whereas exposure to 0.5 µg/ml had no effect (Fig. S2).

### Glucocorticoid Receptor Antagonist RU486 Abolished the Decreased Immune Response Induced by BPA

**Figure 5:**
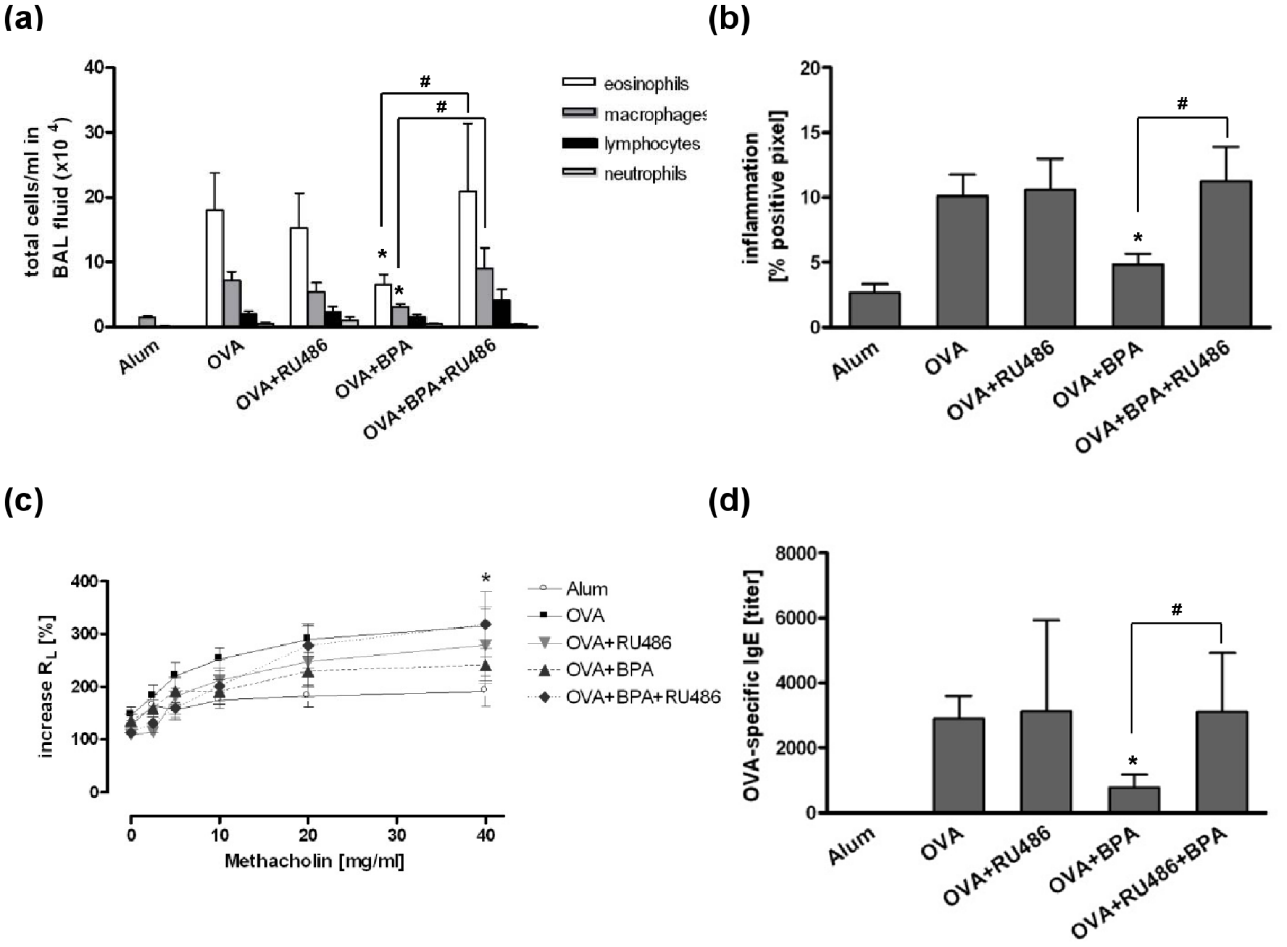
Glucocorticoid receptor antagonist RU486 abolished the decreased immune response induced by BPA. Adult mice were exposed to 5 µg/ml BPA via drinking water. RU486 was given intraperitoneally 3 times/week during OVA-immunisation. Treatment with RU486 reversed the BPA-induced effect on total cell number in BAL fluid (a), lung inflammation (b), lung resistance (c) and OVA-specific IgE serum levels (d). Data are expressed as mean ± SEM, n≥5 animals per group. *P<0.05 OVA and ^#^P<0.05 OVA+BPA+RU486 *vs.* OVA+BPA.

Since it has been shown that BPA can bind to the glucocorticoid receptor (GR) as an agonist [31], [32], we tested whether blocking this receptor would abolish the BPA- induced suppressive effect on the allergic immune response. Treatment of mice with the GR antagonist RU486 reversed the decreased allergic immune response induced by BPA leading to an eosinophilic airway inflammation, AHR and OVA-specific IgE levels comparable to OVA-sensitized control mice (Fig. 5A–D).

## Discussion

Although in recent years several experimental studies have addressed the question, whether BPA may affect the development and the outcome of allergic diseases, the overall picture did not become clearer. Whereas some studies showed a BPA-induced increase of Th2-driven immune reactions with enhanced IgE and IL-4 cytokine levels in adult mice [33], [34], others could demonstrate an augmentation of Th1-mediated responses with rather decreased IgE levels [14], [15]. In further studies, maternal BPA exposure came into focus, since it has been suggested that the prenatal period represents a critical window in which the developing immune system may be primed towards a specific immune phenotype [11]. However, the data concerning prenatal BPA exposure are diverse, ranging from increased asthma susceptibility [12], [35] to an elevated Th1 immune response [36] and no effects [37]. These striking differences might be mainly caused by the used BPA doses ranging from 0.5–5000 µg/kg/day and diverse exposure routes resulting in different bioavailability and metabolism of BPA [38]. In the present study, we exposed mice for different time periods during the whole lifespan to 5 µg/ml BPA via the drinking water. This was half of the dose used in experimental mouse models demonstrating an asthma-promoting effect in the offspring after maternal BPA exposure [12], [35]. Assuming a water intake of the mice between 3 and 5 ml/day [39] the BPA dose/day was about 15–25 µg. This is 10 to 20 times higher than the “No Observed Adverse Effect Level” (NOAEL, 50 µg/kg/day). However, the concentration detected in serum of adult mice in this study was comparable to detected BPA levels of up to 19 ng/ml in maternal serum [40]. Despite using the limited ELISA method in comparison to HPLC-mass spectrometry, we could also find elevated BPA serum levels in pups nursed by exposed mothers, which is in line with human studies showing that BPA may cross the placental barrier and can also be detected in breast milk [6]. However, in the present study we could not observe an effect on the asthma outcome in the offspring after maternal BPA exposure, although a trend was detectable in particular after perinatal exposure. One could assume that levels twice as high (10 µg/ml) would exert a similar asthma-promoting impact in our model as described by others using a BPA concentration of 10 µg/ml [12], [35]. On the other hand, we speculate that the daily exposure of 30–50 µg BPA (assuming 3–5 ml water intake/day) will probably result in much higher BPA concentrations than the described maximum level of 20 ng/ml in human serum [6].

Whereas no effect was observed after pre- and perinatal BPA exposure, an increased allergic airway inflammation was found when BPA was given from birth until the end of the asthma protocol. Interestingly, similar results were found in a recent study from Donohue *et al.* investigating BPA exposure and asthma development among inner-city children [5]. The authors showed a positive association between postnatal urinary BPA concentrations and wheeze at ages of 5 and 6 years, whereas prenatal BPA exposure was inversely associated with wheeze at age of 5 years. Although this is in line with our results, there are contrary findings from Spanier *et al.* demonstrating an association of prenatal BPA exposure and increased odds of child’s wheeze [3]. Donohue *et al.* explained the differences with the study design assessing BPA exposure during the last trimester, whereas Spanier and colleagues measured BPA exposure during the second trimester. The second trimester encompasses formation of the terminal bronchioli and the initiation of antigen-specific immune responses [41] and therefore, could be a critical period for airway and immune development. In contrast, others have identified the third trimester as a critical window of fetal susceptibility for BPA effects on lung development [42]. However, it is quite unlikely that an exposure to BPA will be limited to such an exact period. Furthermore, this explanation fails in respect to our model since we exposed the mice during the whole gravidity.

Either way, only the developing immune system during early life seems to be susceptible to an asthma-promoting impact of BPA because an exposure to this compound in later life led to opposed effects in our model. However, there are only very limited information about underlying mechanisms explaining the Th2-supporting properties of BPA. Previous studies have demonstrated that pollutants may interfere with IL-12 production leading to a decreased Th1 differentiation [28], [43]. Using murine as well as human DC differentiation cultures we could also observe diminished IL-12 levels in the presence of BPA. This was similar to findings by Guo *et al*., who demonstrated that BPA-exposed DC produced higher levels of IL-10 relative to that of IL-12 and preferentially induced Th2 deviation [16]. However, based on this assumption exposure of the adult mice during antigen sensitization should also enhance the allergic immune response. Though, BPA exposure at this time period resulted in a completely different outcome. Here, BPA induced a significant attenuation of eosinophilic airway inflammation, AHR and antigen-specific IgE levels.

There are several mouse studies demonstrating an increased Th1 reactivity [14], [15], whereas we could not find any signs for a modified Th1/Th2 balance. To clarify, whether BPA might induce a worsened disease outcome at higher concentrations adult mice were exposed to 25 µg/ml. However, even at this BPA dose the allergic airway inflammation was diminished. Since we found no evidence for a Th1/IFN-γ-driven suppression of the allergic immune response as reported before [44], [45], we investigated a possible involvement of the previously described BPA-sensitive glucocorticoid receptor [31], [32]. As glucocorticoids belong to the standard therapy of asthmatics and also reduce asthma parameters in murine asthma models [46], we blocked an agonistic interaction of BPA with the glucocorticoid receptor by treatment with the antagonist RU486. The inhibition of this signaling pathway reversed the asthma prevention by BPA. Therefore, an involvement of the glucocorticoid receptor could explain the immune suppressive effects of BPA during antigen sensitization. It should be noted that BPA is also described as an estrogen-receptor agonist [47] and may exert its effects via this pathway. However, information from the experimental studies that have investigated the role of estrogen receptor activation in asthma development is contradictory and showed both Th2 cell stimulating effects and anti-inflammatory properties [47], [48]. Therefore, we did not analyze a possible involvement of the estrogen receptor in our study.

To evaluate the actual risk of BPA exposure for human asthma, in particular by conclusions based on the use of animal models, the comparability of BPA metabolism between human and the respective animal (e.g. mice) has to be considered. Recent studies demonstrated a similarity of BPA pharmacokinetics in rhesus monkeys and mice with a clear relevance for human exposure [20], [49]. Moreover, these studies also suggest that human exposure to BPA may be much higher than previously assumed, namely by identifying important nonfood sources of exposure to this chemical [7], [20]. However, although there are several studies showing alterations after *in utero* exposure to low doses of BPA, e.g. on reproductive organs [50], [51], no effects on asthma development are reported (including our data) after pre- or perinatal exposure to concentrations below 50 µg/kg/day. In contrast, we observed a beneficial effect on asthma development after BPA exposure during adulthood. However, an asthma-promoting effect was detected after a lifelong BPA exposure, which seems much more likely than an exclusive exposure during adulthood. Although using BPA concentrations higher than the NOAEL, the detected serum levels in mice were comparable to levels found in human serum. In summary, our results demonstrate that the impact of BPA on asthma risk is strongly age-dependent and ranges from asthma-reducing effects during adulthood to asthma-promoting effects after lifelong exposure.

## Supporting Information

Figure S1 **BPA exposure during DC differentiation impaired IL-12 production.** BPA was added during differentiation of murine bone marrow-derived dendritic cells (a) and human monocyte-derived dendritic cells (b). IL-12 production was measured 24 hours after maturation with LPS. Data are expressed as mean ± SEM, n≥4. *P<0.05, ***P<0.001 compared to control.
(TIF)

Figure S2 **BPA exposure during OVA-sensitization reduced allergic airway inflammation in a dose-dependent manner.** Adult mice were exposed to BPA via drinking water during OVA-immunisation protocol. BPA exposure reduced total cell number in BAL fluid (a), OVA-specific IgE serum levels (b) and lung resistance (c). Cytokine production from CD4+ lung T cells was not affected (d). Data are expressed as mean ± SEM, n≥7 animals per group. *P<0.05, **P<0.01 of OVA +5 µg/ml BPA and ^#^P<0.05 of OVA +25 µg/ml BPA compared to OVA.
(TIF)

## References

[1] Umetsu DT, McIntire JJ, Akbari O, Macaubas C, DeKruyff RH (2002) Asthma: an epidemic of dysregulated immunity. Nat Immunol 3: 715–720. 12145657 10.1038/ni0802-715

[2] Pearce N, Douwes J (2006) The global epidemiology of asthma in children. Int J Tuberc Lung Dis 10: 125–132. 16499249

[3] Spanier AJ, Kahn RS, Kunselman AR, Hornung R, Xu Y, et al (2012) Prenatal exposure to bisphenol A and child wheeze from birth to 3 years of age. Environ Health Perspect 120: 916–920. 22334053 10.1289/ehp.1104175 PMC3385426

[4] Vaidya SV, Kulkarni H (2012) Association of urinary bisphenol A concentration with allergic asthma: results from the National Health and Nutrition Examination Survey 2005–2006. J Asthma 49: 800–806. 22957848 10.3109/02770903.2012.721041

[5] Donohue KM, Miller RL, Perzanowski MS, Just AC, Hoepner LA, et al (2013) Prenatal and postnatal bisphenol A exposure and asthma development among inner-city children. J Allergy Clin Immunol 131: 736–742. 23452902 10.1016/j.jaci.2012.12.1573 PMC3643970

[6] Vandenberg LN, Hauser R, Marcus M, Olea N, Welshons WV (2007) Human exposure to bisphenol A (BPA). Reprod Toxicol 24: 139–177. 17825522 10.1016/j.reprotox.2007.07.010

[7] Vandenberg LN, Hunt PA, Myers JP, Vom Saal FS (2013) Human exposures to bisphenol A: mismatches between data and assumptions. Rev Environ Health 28: 37–58. 23612528 10.1515/reveh-2012-0034

[8] Biedermann S, Tschudin P, Grob K (2010) Transfer of bisphenol A from thermal printer paper to the skin. Anal Bioanal Chem 398: 571–576. 20623271 10.1007/s00216-010-3936-9

[9] Rochester JR (2013) Bisphenol A and human health: A review of the literature. Reprod Toxicol 42C: 132–155. 10.1016/j.reprotox.2013.08.008 23994667

[10] Wetherill YB, Akingbemi BT, Kanno J, McLachlan JA, Nadal A, et al (2007) In vitro molecular mechanisms of bisphenol A action. Reprod Toxicol 24: 178–198. 17628395 10.1016/j.reprotox.2007.05.010

[11] Ege MJ, Bieli C, Frei R, van Strien RT, Riedler J, et al (2006) Prenatal farm exposure is related to the expression of receptors of the innate immunity and to atopic sensitization in school-age children. J Allergy Clin Immunol 117: 817–823. 16630939 10.1016/j.jaci.2005.12.1307

[12] Midoro-Horiuti T, Tiwari R, Watson CS, Goldblum RM (2010) Maternal bisphenol a exposure promotes the development of experimental asthma in mouse pups. Environ Health Perspect 118: 273–277. 20123615 10.1289/ehp.0901259 PMC2831929

[13] O’Brien E, Dolinoy DC, Mancuso P (2013) Perinatal bisphenol A exposures increase production of pro-inflammatory mediators in bone marrow-derived mast cells of adult mice. J Immunotoxicol. 10.3109/1547691X.2013.822036 PMC3983174 23914806

[14] Alizadeh M, Ota F, Hosoi K, Kato M, Sakai T, et al (2006) Altered allergic cytokine and antibody response in mice treated with Bisphenol A. J Med Invest. 53: 70–80. 10.2152/jmi.53.70 16537998

[15] Goto M, Takano-Ishikawa Y, Ono H, Yoshida M, Yamaki K, et al (2007) Orally administered bisphenol A disturbed antigen specific immunoresponses in the naive condition. Biosci Biotechnol Biochem 71: 2136–2143. 17827700 10.1271/bbb.70004

[16] Guo H, Liu T, Uemura Y, Jiao S, Wang D, et al (2010) Bisphenol A in combination with TNF-alpha selectively induces Th2 cell-promoting dendritic cells in vitro with an estrogen-like activity. Cell Mol Immunol 7: 227–234. 20383177 10.1038/cmi.2010.14 PMC4002911

[17] Inadera H, Sekiya T, Yoshimura T, Matsushima K (2000) Molecular analysis of the inhibition of monocyte chemoattractant protein-1 gene expression by estrogens and xenoestrogens in MCF-7 cells. Endocrinology 141: 50–59. 10614622 10.1210/endo.141.1.7233

[18] Yan H, Takamoto M, Sugane K (2008) Exposure to Bisphenol A prenatally or in adulthood promotes T(H)2 cytokine production associated with reduction of CD4CD25 regulatory T cells. Environ Health Perspect 116: 514–519. 18414636 10.1289/ehp.10829 PMC2290985

[19] Epstein MM (2004) Do mouse models of allergic asthma mimic clinical disease? Int Arch Allergy Immunol 133: 84–100. 14726635 10.1159/000076131

[20] Taylor JA, Vom Saal FS, Welshons WV, Drury B, Rottinghaus G, et al (2011) Similarity of bisphenol A pharmacokinetics in rhesus monkeys and mice: relevance for human exposure. Environ Health Perspect 119: 422–430. 20855240 10.1289/ehp.1002514 PMC3080921

[21] Fund TBC (2010) Safer Alternatives to Bisphenol A (BPA). 1–4 p.

[22] Polte T, Hennig C, Hansen G (2008) Allergy prevention starts before conception: maternofetal transfer of tolerance protects against the development of asthma. J Allergy Clin Immunol 122: 1022–1030 e1025. 10.1016/j.jaci.2008.09.014 19000583

[23] Polte T, Hansen G (2008) Maternal tolerance achieved during pregnancy is transferred to the offspring via breast milk and persistently protects the offspring from allergic asthma. Clin Exp Allergy 38: 1950–1958. 18778271 10.1111/j.1365-2222.2008.03096.x

[24] Reiprich M, Rudzok S, Schutze N, Simon JC, Lehmann I, et al (2013) Inhibition of endotoxin-induced perinatal asthma protection by pollutants in an experimental mouse model. Allergy 68: 481–489. 23409786 10.1111/all.12121

[25] Polte T, Foell J, Werner C, Hoymann HG, Braun A, et al (2006) CD137-mediated immunotherapy for allergic asthma. J Clin Invest 116: 1025–1036. 16528411 10.1172/JCI23792 PMC1395480

[26] Polte T, Jagemann A, Foell J, Mittler RS, Hansen G (2007) CD137 ligand prevents the development of T-helper type 2 cell-mediated allergic asthma by interferon-gamma-producing CD8+ T cells. Clin Exp Allergy 37: 1374–1385. 17845419 10.1111/j.1365-2222.2007.02785.x

[27] Polte T, Behrendt AK, Hansen G (2006) Direct evidence for a critical role of CD30 in the development of allergic asthma. J Allergy Clin Immunol 118: 942–948. 17030250 10.1016/j.jaci.2006.07.014

[28] Schutze N, Lehmann I, Bonisch U, Simon JC, Polte T (2010) Exposure to mycotoxins increases the allergic immune response in a murine asthma model. Am J Respir Crit Care Med 181: 1188–1199. 20194814 10.1164/rccm.200909-1350OC

[29] Bickert T, Trujillo-Vargas CM, Duechs M, Wohlleben G, Polte T, et al (2009) Probiotic Escherichia coli Nissle 1917 Suppresses Allergen-Induced Th2 Responses in the Airways. Int Arch Allergy Immunol 149: 219–230. 19218814 10.1159/000199717

[30] Polte T, Fuchs L, Behrendt AK, Hansen G (2009) Different role of CD30 in the development of acute and chronic airway inflammation in a murine asthma model. Eur J Immunol 39: 1736–1742. 19544310 10.1002/eji.200839004

[31] Prasanth GK, Divya LM, Sadasivan C (2010) Bisphenol-A can bind to human glucocorticoid receptor as an agonist: an in silico study. J Appl Toxicol 30: 769–774. 20669259 10.1002/jat.1570

[32] Sargis RM, Johnson DN, Choudhury RA, Brady MJ (2010) Environmental endocrine disruptors promote adipogenesis in the 3T3-L1 cell line through glucocorticoid receptor activation. Obesity (Silver Spring) 18: 1283–1288. 19927138 10.1038/oby.2009.419 PMC3957336

[33] Lee MH, Chung SW, Kang BY, Park J, Lee CH, et al (2003) Enhanced interleukin-4 production in CD4+ T cells and elevated immunoglobulin E levels in antigen-primed mice by bisphenol A and nonylphenol, endocrine disruptors: involvement of nuclear factor-AT and Ca2+. Immunology 109: 76–86. 12709020 10.1046/j.1365-2567.2003.01631.x PMC1782943

[34] Tian X, Takamoto M, Sugane K (2003) Bisphenol A promotes IL-4 production by Th2 cells. Int Arch Allergy Immunol 132: 240–247. 14646385 10.1159/000074305

[35] Nakajima Y, Goldblum RM, Midoro-Horiuti T (2012) Fetal exposure to bisphenol A as a risk factor for the development of childhood asthma: an animal model study. Environ Health 11: 8. 22353195 10.1186/1476-069X-11-8 PMC3306825

[36] Yoshino S, Yamaki K, Li X, Sai T, Yanagisawa R, et al (2004) Prenatal exposure to bisphenol A up-regulates immune responses, including T helper 1 and T helper 2 responses, in mice. Immunology 112: 489–495. 15196218 10.1111/j.1365-2567.2004.01900.x PMC1782504

[37] Bauer SM, Roy A, Emo J, Chapman TJ, Georas SN, et al (2012) The effects of maternal exposure to bisphenol A on allergic lung inflammation into adulthood. Toxicol Sci 130: 82–93. 22821851 10.1093/toxsci/kfs227 PMC3621363

[38] Pottenger LH, Domoradzki JY, Markham DA, Hansen SC, Cagen SZ, et al (2000) The relative bioavailability and metabolism of bisphenol A in rats is dependent upon the route of administration. Toxicol Sci 54: 3–18. 10746927 10.1093/toxsci/54.1.3

[39] Bachmanov AA, Reed DR, Beauchamp GK, Tordoff MG (2002) Food intake, water intake, and drinking spout side preference of 28 mouse strains. Behav Genet 32: 435–443. 12467341 10.1023/a:1020884312053 PMC1397713

[40] Schonfelder G, Wittfoht W, Hopp H, Talsness CE, Paul M, et al (2002) Parent bisphenol A accumulation in the human maternal-fetal-placental unit. Environ Health Perspect 110: A703–707. 12417499 10.1289/ehp.110-1241091 PMC1241091

[41] Jeffrey PK (1998) The development of large and small airways. Am J Respir Crit Care Med 157: S174–180. 9606315 10.1164/ajrccm.157.5.rsaa-1

[42] Van Winkle LS, Murphy SR, Boetticher MV, VandeVoort CA (2013) Fetal exposure of rhesus macaques to bisphenol a alters cellular development of the conducting airway by changing epithelial secretory product expression. Environ Health Perspect 121: 912–918. 23757601 10.1289/ehp.1206064 PMC3734491

[43] Bönisch U, Böhme A, Kohajda T, Mögel I, Schütze N, et al (2012) Volatile Organic Compounds Enhance Allergic Airway Inflammation in an Experimental Mouse Model. PLoS One 7: e39817. 22802943 10.1371/journal.pone.0039817 PMC3389035

[44] Li XM, Chopra RK, Chou TY, Schofield BH, Wills-Karp M, et al (1996) Mucosal IFN-gamma gene transfer inhibits pulmonary allergic responses in mice. J Immunol 157: 3216–3219. 8871613

[45] Lack G, Bradley KL, Hamelmann E, Renz H, Loader J, et al (1996) Nebulized IFN-gamma inhibits the development of secondary allergic responses in mice. J Immunol 157: 1432–1439. 8759723

[46] De Bie JJ, Hessel EM, Van Ark I, Van Esch B, Hofman G, et al (1996) Effect of dexamethasone and endogenous corticosterone on airway hyperresponsiveness and eosinophilia in the mouse. Br J Pharmacol 119: 1484–1490. 8968559 10.1111/j.1476-5381.1996.tb16062.x PMC1915832

[47] Cai Y, Zhou J, Webb DC (2012) Estrogen stimulates Th2 cytokine production and regulates the compartmentalisation of eosinophils during allergen challenge in a mouse model of asthma. Int Arch Allergy Immunol 158: 252–260. 22398379 10.1159/000331437

[48] Regal JF, Fraser DG, Weeks CE, Greenberg NA (2000) Dietary phytoestrogens have anti-inflammatory activity in a guinea pig model of asthma. Proc Soc Exp Biol Med 223: 372–378. 10721007 10.1046/j.1525-1373.2000.22353.x

[49] Doerge DR, Twaddle NC, Vanlandingham M, Fisher JW (2011) Pharmacokinetics of bisphenol A in neonatal and adult CD-1 mice: inter-species comparisons with Sprague-Dawley rats and rhesus monkeys. Toxicol Lett 207: 298–305. 21983029 10.1016/j.toxlet.2011.09.020

[50] Nagel SC, vom Saal FS, Thayer KA, Dhar MG, Boechler M, et al (1997) Relative binding affinity-serum modified access (RBA-SMA) assay predicts the relative in vivo bioactivity of the xenoestrogens bisphenol A and octylphenol. Environ Health Perspect 105: 70–76. 9074884 10.1289/ehp.9710570 PMC1469837

[51] Welshons WV, Nagel SC, Thayer KA, Judy BM, Vom Saal FS (1999) Low-dose bioactivity of xenoestrogens in animals: fetal exposure to low doses of methoxychlor and other xenoestrogens increases adult prostate size in mice. Toxicol Ind Health 15: 12–25. 10188188 10.1177/074823379901500103

