# Supplementary figures and images for: Lifetime-Dependent Effects of Bisphenol A on Asthma Development in an Experimental Mouse Model
Source: PLoS One. 2014 Jun 20;9(6):e100468. doi: 10.1371/journal.pone.0100468 (PMC4065062; doi:10.1371/journal.pone.0100468)

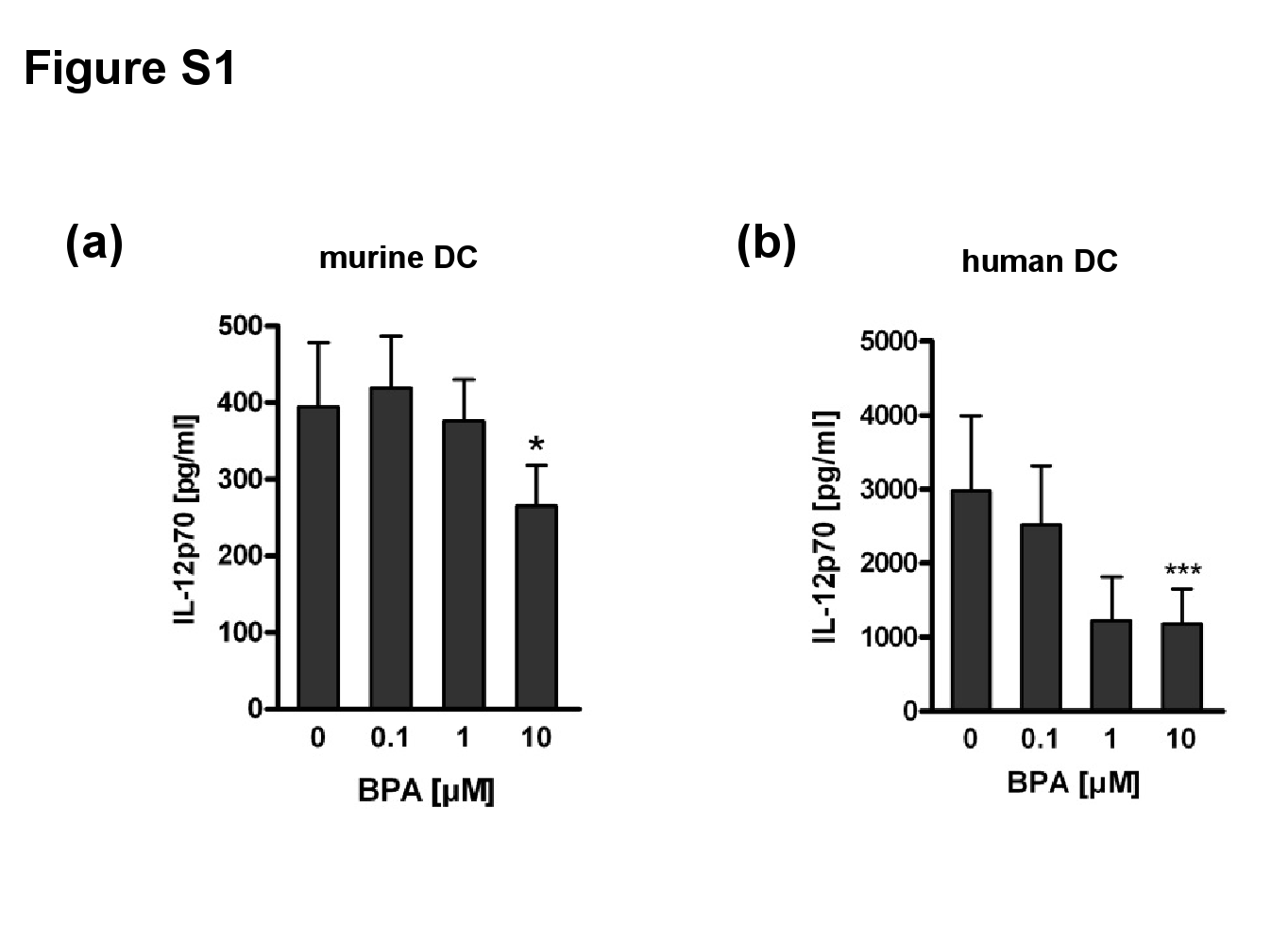

Supplement: Figure S1 — BPA exposure during DC differentiation impaired IL-12 production. BPA was added during differentiation of murine bone marrow-derived dendritic cells (a) and human monocyte-derived dendritic cells (b). IL-12 production was measured 24 hours after maturation with LPS. Data are expressed as mean ± SEM, n≥4. *P<0.05, ***P<0.001 compared to control. (TIF) [file pone.0100468.s001.tif]

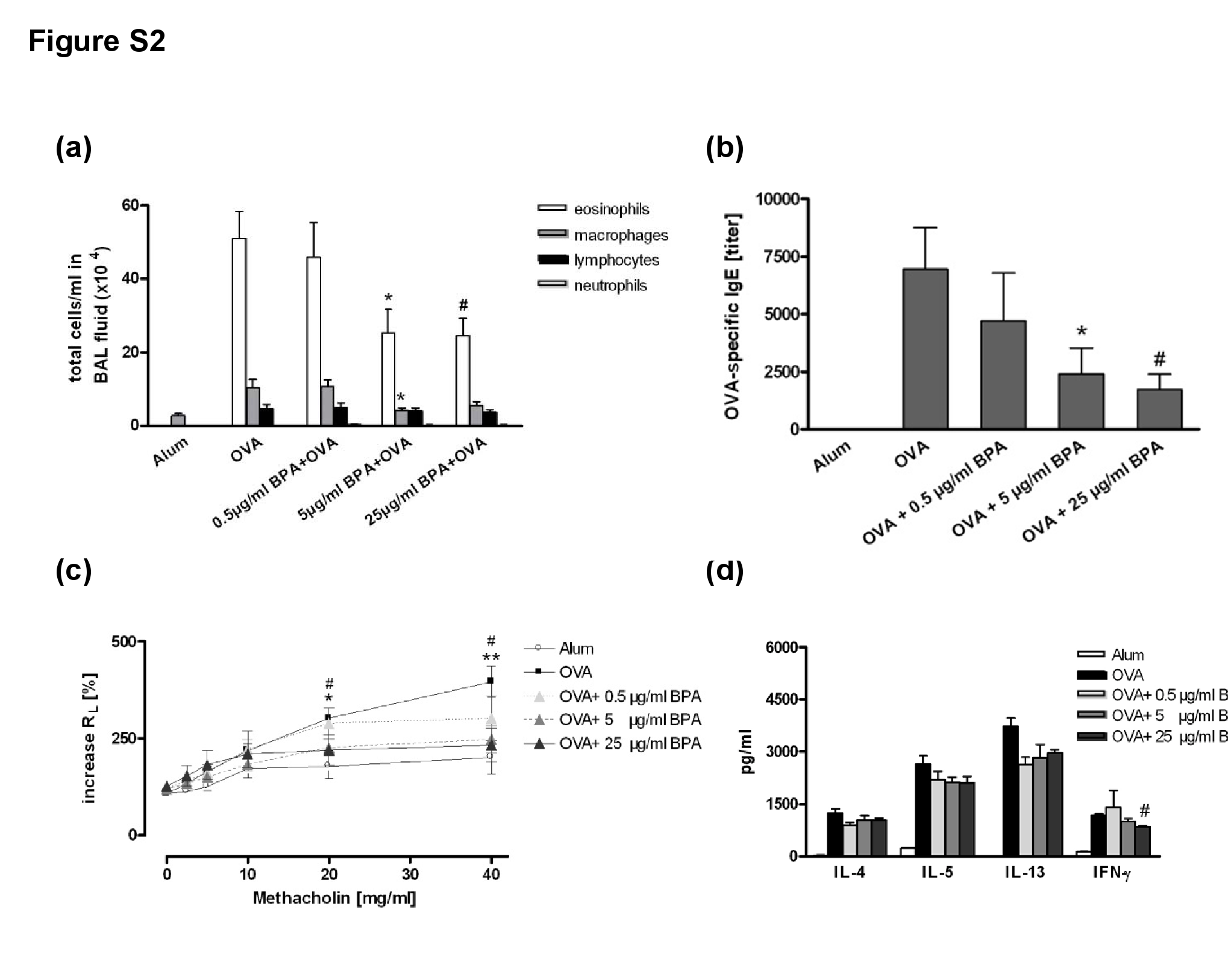

Supplement: Figure S2 — BPA exposure during OVA-sensitization reduced allergic airway inflammation in a dose-dependent manner. Adult mice were exposed to BPA via drinking water during OVA-immunisation protocol. BPA exposure reduced total cell number in BAL fluid (a), OVA-specific IgE serum levels (b) and lung resistance (c). Cytokine production from CD4+ lung T cells was not affected (d). Data are expressed as mean ± SEM, n≥7 animals per group. *P<0.05, **P<0.01 of OVA +5 µg/ml BPA and #P<0.05 of OVA +25 µg/ml BPA compared to OVA. (TIF) [file pone.0100468.s002.tif]
